# Supplementary material for: Protocol for development and validation of instruments to measure women’s empowerment in urban sanitation across countries in South Asia and Sub-Saharan Africa: the Agency, Resources and Institutional Structures for Sanitation-related Empowerment (ARISE) scales
Source: BMJ Open. 2022 Feb 16;12(2):e053104. doi: 10.1136/bmjopen-2021-053104 (PMC8860033; doi:10.1136/bmjopen-2021-053104)
Supplement: Supplementary data [file bmjopen-2021-053104supp001.pdf]

Supplementary Table 1. Instruments to measure empowerment identified through a landscape analysis

Name of Tool	Citation
Adherence to Traditional Gender Relations Scale	Bogale, B., Wondafrash, M., Tilahun, T., & Girma, E. (2011). Married women's decision making power on modern contraceptive use in urban and rural southern Ethiopia. *Public Health, 11*, 1-7.
Adolescents' Attitudes Towards Gender Role Scale	Zuo, X., Lou, C., Gao, E., Cheng, Y., Niu, H., & Zabin, L.S. (2012). Gender differences in adolescent premarital sexual permissiveness in three Asian cities: Effects of gender-role attitudes. *Journal of Adolescent Health, 50*, S18-S25.
Adolescents' Attitude toward Women's Empowerment in Household Decisions Index	Gomez, A.M., Speizer, I., & Moracco, K.E. (2011). Linkages between gender equity and intimate partner violence among urban Brazilian youth. *Journal of Adolescent Health, 49*(4), 393-399.
Assessment of Intimate Partner Violence	What Works to Prevent Violence Against Women and Girls Global Programme. (2015). *Standard Outcomes for Assessment of Intimate Partner Violence*. Pretoria, South Africa: What Works to Prevent Violence Against Women and Girls Global Programme.
Assessment of Non-Partner Sexual Violence	Jadhav, A., Weitzman, A., & Smith-Greenaway, E. (2016) Household sanitation facilities and women's risk of non-partner sexual violence in India. *BMC Public Health, 16*(1).
Collective Efficacy Scales	Delea, Maryann G., Gloria D. Sclar, Mulat Woreta, Regine Haardörfer, Corey L. Nagel, Bethany A. Caruso, Robert Dreibelbis, Abebe G. Gobezayehu, Thomas F. Clasen, and Matthew C. Freeman. "Collective efficacy: development and validation of a measurement scale for use in public health and development programmes." *International journal of environmental research and public health* 15, no. 10 (2018): 2139.
Compendium of Gender Scales	Nanda, G. (2011). *Compendium of Gender Scales*. Washington, DC: FHI 360/C-Change.
Control Over Finances Scale	Anderson, L., Stahley, K., & Cullen, A. (2014). Individual and intra-household positionality in Vietnam. *Journal of Behavioral and Experimental Economics, 49*, 26-34.
Control Over Women's Income	Mahmud, S., Shah, N.M., & Becker, S. (2012). Measurement of Women's Empowerment in Rural Bangladesh. *World Development, 40*(3), 610-619.
Decision Making Index (DMI)	Stephenson, R. (2010). Community-level gender equity and extramarital sexual risk-taking among married men in eight African countries. *International Perspectives on Sexual and Reproductive Health, 36*(4), 178-188.

Decision-making Autonomy Index	Mahmood, N. (2002). Women's role in domestic decision-making in Pakistan: Implications for reproductive behavior. *Pakistan Development Review, 41*(2), 121-148.
Decision-making Measure	Bui, T. C., Diamond, P.M., Markham, C., Ross, M.W., Nguyen-Le, T.A., &Tran, L.H. (2010). Gender relations and sexual communication among female students in the Mekong River Delta of Vietnam. *Culture, Health & Sexuality, 12*(6), 591-601.
DHS Women's Status Module	The Demographic and Health Surveys Program, USAID. (2020). Demographic and Health Surveys: Model Woman's Questionnaire. Washington, DC: USAID.
Economic Freedom Measure	Corroon, M., Speizer, I.S., Fotso, J.C., Akiode, A., Saad, A., Calhoun, L., & Irani, L. (2014). The role of gender empowerment on reproductive health outcomes in urban Nigeria. *Maternal and Child Health Journal, 18*, 307-315.
Economic Power Index	Morgan, S., P. (2002). Muslim and non-Muslim differences in female autonomy and fertility: Evidence from four Asian countries. *Population and Development Review, 28*(3), 515-537.
Economic Security Scale	Al-Mamun, A., Wahab, S.A., Mazumder, M.N.H., & Su, Z. (2014). Empirical investigation on the impact of microcredit on women empowerment in urban peninsular Malaysia. *The Journal of Developing Areas, 48.*
Education for Women Community Norm Measure	Dynes, M., Stephenson, R., Rubardt, M., & Bartel, d. (2012). The influence of perceptions of community norms on current contraceptive use among men and women in Ethiopia and Kenya. *Health & Place, 18*(4), 766-773.
Equity for Girls Scale	Waszak, C., Severy, L.J., Kafafi, L., & Badawi, I. (2001). Fertility behavior and psychological stress: The mediating influence of gender norm beliefs among Egyptian women. *Psychology of Women Quarterly, 25,* 197-208.
Financial Autonomy Scale (DHS)	Mumtaz, Z., Slaymaker, E., & Salway, S. (2005). *Condom Use in Uganda and Zimbabwe: Exploring the Influence of Gendered Access to Resources and Couple-Level Dynamics*. Calverton, MD: ORC Macro, MEASURE DHS.
Freedom of Movement Outside the Home Scale (DHS)	Mogford, E. (2011). When status hurts: dimensions of women's status and domestic abuse in rural Northern India. *Violence Against Women, 17*(7), 835-857.
Freedom of Movement Scale	Ghuman, S.J. (2003). Women's autonomy and child survival: A comparison of Muslims and non-Muslims in four Asian countries. *Demography, 40*(3), 419-436.
Gender Equality Scale	Do, M., & Kurimoto, N. (2012). Women's empowerment and choice of contraceptive methods in selected African countries. *International Perspectives on Sexual and Reproductive Health, 3*8(1), 23-33.

Gender Equitable Men Scale (GEM)	Morgan, S.P. (2002). Muslim and non-Muslim differences in female autonomy and fertility: Evidence from four Asian countries. P*opulation and Development Review, 28*(3), 515-537.
Gender Equity Scale	Agrawal, A., Bloom, S.S., Suchindran, C., Curtis, S., & Angeles, G. (2014). Gender-based power and couples' HIV risk in Uttar Pradesh and Uttarakhand, north India. *International Perspectives on Sexual and Reproductive Health, 40*(4), 196-205.
Gender Role Attitudes Questionnaire	Mistry, R., Galal, O., & Lu, M. (2009). Women's autonomy and pregnancy care in rural India: a contextual analysis". *Social Science and Medicine, 69*(6), 926-933.
Gender WASH Monitoring Tool	Leong, L., Nicholson, K., Elkington, D., & Hogan, E. (2014). *Gender and WASH Monitoring Tool*. Melbourne, Australia: PLAN International.
Hopkins Symptom Checklist	Parloff, M.B., Kelman, H.C., & Frank, J.D. (1954). Comfort, effectiveness, and self-awareness as criteria for improvement in psychotherapy. *American Journal of Psychiatry, 3*, 343-351.
Household Decision-Making Authority Scale	Anderson, L., Stahley, K., & Cullen, A. (2014). Individual and intra-household positionality in Vietnam. *Journal of Behavioral and Experimental Economics, 49,* 26-34.
Household Survey to Assess Sanitation Decision Making	Routray,P., Torondel, B., Clasen, T., & Schmidt, W. (2017). Women's role in sanitation decision making in rural coastal Odisha, India. *PLoS ONE, 12*(5).
Household Water InSecurity Experiences (HWISE) Scale	Young, S.L., Boateng, G.O., Jamaluddine, Z. Miller, J.D., Frongillo, E.A., Neilands, T.B., Collins, S.M., Wutich, A., Jepson, W.E., & Stoler, J. (2019). The Household Water InSecurity Experiences (HWISE) scale: Development and validation of a household water inseucirty measure for low-income and middle-income countries. *BMJ Global Health, 4*.
IASC: Guidelines for Integrating Gender-Based Violence Interventions in Humanitarian Action – 2015	Inter-Agency Standing Committee (IASC). (2015). *Guidelines for Integrating Gender-Based Violence Interventions in Humanitarian Action: Reducing Risk, Promoting Resilience and Aiding Recovery*. IASC.
Individual Deprivation Measure	Bessell, S. (2015). The Individual Deprivation Measure: Measuring poverty as if gender and inequality matter. *Gender & Development, 23*(2), 223-240.
Item on Neighborhood Water Quality	Siddiqui, R.N., & Pandey, J. (2003). Coping with environmental stressors by urban slum dwellers. *Environment and Behavior*, *35(5),* 589-604.
Joint Monitoring Programme (JMP) for Water Supply, Sanitation, and Hygiene – 2018	United Nations Children's Fund (UNICEF), & World Health Organization (WHO). (2018). *Core Questions on Water, Sanitation and Hygiene for Household Surveys*. New York, NY: UNICEF & WHO.
LSHTM Household WASH Access Questionnaire	London School of Hygiene and Tropical Medicine (LSHTM). (2014). *Malawi WASH and Disability Survey 2014: Household WASH Access Questionnaire*. London, United Kingdom: LHSTH.

Measuring Gender-Transformative Change	Hillenbrand, E., Karim, N., Mohanraj, P., & Wu, D. (2015). *Measuring Gender-Transformative Change: A Review of Literature and Promising Practices.* Atlanta, GA: CARE USA.
Men's Household Decision-making Power	Weziak-Bialowolska, D. (2015). Differences in gender norms between countries: Are they valid? The issue of measurement invariance. *European Journal of Population, 31*, 51-76.
Menstrual Hygiene Management in Nepal	Morrison, J., Basnet, M., Bhatta, A., Khimbajar, S., Joshi, D., & Baral, S. (2016). *Menstrual Hygiene Managemenet in Udaypur and Sindhuli Districts of Nepal.* London, UK: WaterAid.
Menstrual Hygiene Management Operational Guidelines	Haver, J., & Long, J.L. (2015). *Menstrual Hygiene Management: Operational Guidelines.* Fairfield, CT: Save the Children.
Mobility Scale	Zuo, X., Lou, C., Gao, E., Cheng, Y., Niu, H., & Zabin, L. S. (2012). Gender differences in adolescent premarital sexual permissiveness in three Asian cities: Effects of gender-role attitudes. *Journal of Adolescent Health, 50*, S18-S25.
Oxfam: A "How to" Guide to Measuring Women's Empowerment – 2017	Lombardini, S., Bowman, K., & Garwood, R. (2017). *A 'How To' Guide to Measuring Women's Empowerment: Sharing Experience from Oxfam's Impact Evaluations*. Cowley, UK: Oxfam GB.
Patriarchal Beliefs Scale	Yoon, E., Adams, K., Hogge, I., Bruner, J.P., Surya, S., & Bryant, F.B. (2015). Development and validation of the Patriarchal Beliefs Scale. *Journal of Counseling Psychology, 62*(2), 264-279.
Permission to Go Out Scale	Al-Riyami, A. A., & Afifi, M. (2003). Determinants of women's fertility in Oman. *Saudi Medical Journal, 24*(7), 748-753.
Project-level Women's Empowerment in Agriculture Index (pro-WEAI)	Malapit, H., Quisumbing, A., Meinzen-Dick, R., Seymour, G., Martinez, E.M., Heckert, J., Rubin, D., Vaz, A., & Yount, K.M. (2019). Development of the project-level Women's Empowerment in Agriculture Index (pro-WEAI). *World Development, 122*, 675-692.
Resources and Constraints in Marriage	Mahmood, N. (2002) Women's role in domestic decision-making in Pakistan: Implications for reproductive behaviour. *Pakistan Development Review, 41*(2), 121-148
Restrictions on Wife's Mobility	Mishra, A., Nanda, P., Speizer, I. S., Calhoun, L. M., Zimmerman, A., & Bhardwaj, R. (2014). Men's attitudes on gender equality and their contraceptive use in Uttar Pradesh India. *Reproductive Health*, *11*.
Rights and Privileges of Males Scale	Donta, B., Nair, S., Begum, S., & Prakasam, C.P. (2015). Association of domestic violence from husband and women empowerment in slum community, Mumbai. *Journal of Interpersonal Violence*, 1-13.

Sanitation Insecurity Measure	Caruso, B.A., Clasen, T., Yount, K.M., Cooper, H.L.F., Hadley, C., & Haardorfer, R. (2017). Assessing women's negative sanitation experiences and concerns: The development of a novel sanitation insecurity measures. *International Journal of Environmental Research and Public Health, 14*(7).
Survey-based Women's Empowerment Index (SWPER)	Ewerling, F., Lynch, J.W., Victora, C.G., van Eerdewijk, A., Tyszler, M., & Barros, A.J.D. (2017). The SWPER index for women's empowerment in Africa: Development and validation of an index based on survey data. *The Lancet Global Health, 5*(9), 916-923.
Traditional Gender Role Ideology Index	Firestone, J.M., Harris, R.J., & Vega, W.A. (2003). The impact of gender role ideology, male expectancies, and acculturation on wife abuse. *International Journal of Law and Psychiatry, 25*(3), 549-564.
Understanding and Measuring Women's Economic Empowerment	Golla, A.M., Malhotra, A., Nanda, P., & Mehra, R. (2018). *Understanding and Measuring Women's Economic Empowerment: Definition, Framework, and Indicators*. Washington, DC: International Center for Research on Women.
WASH Needs Among Female-Headed Households Survey	Carolini, G.Y. (2012). Framing water, sanitation, and hygiene needs among female-headed households in periurban Maputo, Mozambique. *American Journal of Public Health, 102*(2), 256-261.
Water and Development Alliance (WADA)	Water & Development Alliance (WADA), Ipsos, USAID, & Coca Cola. (2018). *Women & Water: A Ripple Effect*. Washington, DC: USAID.
WE-MEASR (Women's Empowerment Multidimensional Evaluation of Agency, Social Capital, & Relations)	CARE USA. (2013). WE-MEASR: A New Tool for Measuring Women's Empowerment in Health Programs (Technical Update) (p. 2). Atlanta, GA: CARE USA Headquarters.
WHO Self-Reporting Questionnaire	Beusenberg, M., & Orley, J. (1994). *A User's Guide to the Self-Reporting Questionnaire*. Geneva, Switzerland: WHO.
WHOQOL-BREF	World Health Organization (WHO) Programme on Mental Health. (1996). *WHOQOL-BREF: Introduction, Administration, Scoring, and Generic Version of the Assessment: Field Trial Version*. Geneva, Switzerland: WHO.
Wife's Autonomy Scale	Mistry, R., Galal, O., & Lu, M. (2009). Women's autonomy and pregnancy care in rural India: A contextual analysis. *Social Science and Medicine*, 69(6), 926-933.
Women's Empowerment in Agriculture Index (WEAI)	Alkire, S., Meinzen-Dick, R. Peterman, A., Quisumbing, A., Seymour, G., & Vaz, A. The Women's Empowerment in Agriculture Index. *Journal of World Development,* 52, 71-91.
Women's Empowerment Index (WEI)	The Hunger Project. (2015). *The Women's Empowerment Index*. New York, NY: The Hunger Project.

Women's Freedom of Movement Scale	Dynes, M., Stephenson, R., Rubardt, M., & Bartel, D. (2012). The influence of perceptions of community norms on current contraceptive use among men and women in Ethiopia and Kenya. *Health & Place, 18*(4), 766-773.
Women's Health-seeking Behaviors Measure	Yount, K., & Li, L. (2010). Domestic violence against married women in Egypt. *Sex Roles, 63*, 332-347.
Women's Household Decision-Making Power Scale	Cunningham, K., Ploubidis, G.B., Menon, P., Ruel, M., Kadiyala, S., Uauy, R., & Ferguson, E. (2015). Women's empowerment in agriculture and child nutritional status in rural Nepal. *Public Health Nutrition, 18*(7), 3134-3145.
