# Supplementary material for: Protocol for development and validation of instruments to measure women’s empowerment in urban sanitation across countries in South Asia and Sub-Saharan Africa: the Agency, Resources and Institutional Structures for Sanitation-related Empowerment (ARISE) scales
Source: BMJ Open. 2022 Feb 16;12(2):e053104. doi: 10.1136/bmjopen-2021-053104 (PMC8860033; doi:10.1136/bmjopen-2021-053104)
Supplement: Supplementary data [file bmjopen-2021-053104supp002.pdf]

Supplementary Table 2. Validity measures for each sub-domain of empowerment

Sub-domain	Existing Items for Validity	Additional Questions/Measures for Validity	# of Items Added	Validity Type	Validity Subtype
**Agency**					
Decision-Making	Index documenting women influencing and making decision about sanitation in the home<br><br>Index documenting women influencing and making decision about sanitation outside of the home	General decision making questions:<br>1. Do you feel comfortable expressing your opinion at a community meeting where only women are present?<br>2. Do you feel comfortable expressing your opinion at a community meeting where both men and women are present?	5	Construct	Convergent
Leadership	Index of participation in leadership positions<br><br>Index of women's assumption of and participation in formal and informal leadership positions	Empowerment Scale in Community Organizing Emotional empowerment dimension, subscale 1 (5-point agree-disagree response options):<br><br>1. I am often a leader in groups.<br>2. I would prefer to be a leader rather than a follower.<br>3. I would rather someone else took over the leadership role when I'm involved in a group project.	3	Construct (Index)<br>Criterion (Empowerment scale)	Convergent<br>Concurrent
Collective Action	Index of women's participation in collective action around sanitation	Community Capacity Index (5 questions)	5	Construct (Index)<br>Criterion (Empowerment scale)	Convergent<br>Concurrent
Freedom of movement	n/a	IHDS-2: Gender Relations: Mobility measure, first subscale (0=no, 1=must inform, 2=yes)<br>please tell us whether you have to ASK PERMISSION of your husband or a senior family member to go…<br><br>to the local health center?<br>to the home of relatives or friends [in the village / neighborhood]?<br>a short distance by train or bus?	3	Criterion	Concurrent
**Resources**					
Bodily Integrity	Demographics questions on privacy of sanitation location		0	Construct (demographic questions)	Convergent
Health	n/a	PROMIS global health subscale ratings	10	Criterion	Concurrent

		1. In general, how would you rate your physical health? (Excellent, Very Good, Good, Fair, Poor)<br><br>WHO 5 Well-being index (5 questions)<br><br>Patient Health Questionnaire - 4 (PHQ 4) (4 questions)			
Safety and Security	Index of "I know a woman in my community who has..."	n/a	0	Construct	Convergent
Privacy	Demographics questions on privacy of sanitation location	n/a	0	Construct (demographic questions)	Convergent
Critical Consciousness	n/a	GSE - 6 (1-4; not at all true, hardly true, moderately true, exactly true):<br><br>1. If someone opposes me, I can find the means and ways to get what I want.<br>2. It is easy for me to stick to my aims and accomplish my goals.<br>3. Thanks to my resourcefulness, I know how to handle unforeseen situations.<br>4. I can solve most problems if I invest the necessary effort.<br>5. I can remain calm when facing difficulties because I can rely on my coping abilities.<br>6. I can usually handle whatever comes my way.	6	Criterion	Concurrent (self-efficacy only, not getting at ability to identify/question gender equity)
Financial and Productive Assets	Demographics questions on asset ownership	When you want or need to buy things like food or clothing for yourself or your family, which of the following answers best describes your situation? (0/1)<br><br>You have your own money so you can usually buy what you need<br>You occasionally have to get the money from your husband or someone else in the household<br>You always have to get the money from your husband or someone else in the household<br>Don't know<br>Not applicable	1	Construct (demographic questions)<br>Criterion (Additional question)	Convergent<br>Concurrent
Time	Questions on water collection (time to collect and frequency of collection) and sanitation (time to walk to a sanitation location)	n/a	0	Construct (demographic questions)	Convergent

Social Capital	n/a	MPSS (5-point agree-disagree response options):<br><br>1. My family really tries to help me.<br>2. I get the emotional help and support I need from my family.<br>3. My friends really try to help me.<br>4. I can count on my friends when things go wrong.<br>5. I can talk about my problems with my family.<br>6. I have friends with whom I can share my joys and sorrows.<br>7. My family is willing to help me make decisions.<br>8. I can talk about my problems with my friends.	8	Criterion	Concurrent
Knowledge and Skills	Demographic questions on schooling attainment; media exposure (through reading a newspaper/magazine, listening to radio, or watching television); and using the Internet	n/a	0	Construct (demographic questions)	Convergent
**Institutional Structures**					
Norms	n/a	Gender equity scale (5-point agree-disagree response options):<br><br>1. A woman can talk to men other than her husband<br>6. A woman can participate in community activities if she wishes to<br>7. The status of women is lower than that of men<br>9. A woman should finish all the household work before taking rest	4	Criterion	Concurrent
Relations	n/a	Brief Family Relationship Scale:<br><br>7. In our family there is a feeling of togetherness.<br>8. In our family we sometimes tell each other about our personal problems.<br>9. In our family we lose our tempers a lot. ®	3	Criterion	Concurrent
